# Mathematical modelling and compensation strategies for printing dot gain

**DOI:** 10.1371/journal.pone.0334921

**Published:** 2025-10-30

**Authors:** Xiaoli Liu, Bingzhong Qiu, Ying-Leh Ling

**Affiliations:** 1 General Education Department, Qilu Medical University, Zibo, Shandong, China; 2 Department of Mathematics, Science and Computer, Politeknik Kuching Sarawak, Kuching, Sarawak, Malaysia; IIIT Kurnool: Indian Institute of Information Technology Design and Manufacturing Kurnool, INDIA

## Abstract

In contemporary printing processes, dot gain is a pivotal factor influencing print quality. This phenomenon, characterized by the loss of image details and the potential for chromatic aberration, poses significant challenges to enhancing print quality. Despite extensive research that has been conducted by numerous scholars on dot gain, effective control and correction of this phenomenon in practical printing operations remain an urgent concern. This study utilized newsprint, offset paper, and coated paper as research objects, and employed the least squares method and MATLAB tools to calculate dot gain compensation values through the “coordinate transformation method” and fit the compensation curve of dot gain. The novelty of this research lies in its development of an integrated mathematical modeling approach that combines least squares optimization with coordinate transformation, providing a computationally efficient alternative to traditional inverse function methods. The experimental results demonstrated that the compensation strategy was effective in the mid-tone and dark-tone areas, significantly enhancing printing accuracy and stability. However, in the bright tone area, further optimization of the compensation effect is necessary. The study proposes a dot gain compensation strategy based on the least squares method, providing the printing industry with new ideas and technical support for enhancing printing quality.

## 1 Introduction

In the contemporary printing industry, the quality of printed materials, as a pivotal medium for the dissemination of information, directly impacts the effective communication of information and the reader’s visual experience [1]. The utilization of high-quality printed materials enables not only the accurate conveyance of design intent but also substantially enhances brand image and user satisfaction [2]. In the context of intensifying market competition and escalating consumer expectations for superior quality, printing companies are compelled to pursue continuous technological enhancements to satisfy market demands for high-quality print products [3]. However, “dot gain” has emerged as a pivotal factor influencing printing quality [4]. This phenomenon, characterized by the loss of image details and the potential for color distortion, poses significant challenges to print quality enhancement [5].

Dot gain is defined as the phenomenon of expansion of the area of the dot on the printing plate relative to the original dot area when it is transferred to the substrate material during the printing process [6]. This phenomenon is common in flexographic printing, offset printing, and other common printing processes, especially in areas with high dot values [7].In recent years, many scholars have conducted in-depth research on dot gain. Fang and Wu utilized the least squares method to analyze the dot gain phenomenon of self-adhesive materials in the flexographic printing process and proactively explored the corresponding compensation strategy [8]. Xiao and Ma et al. concentrated on the printing of metal cans and investigated the intrinsic relationship between 3D dot plate-making parameters and dot gain [9]. Li’s study, which focused on inkjet printing, found that dot gain significantly affects color rendering during the printing process on corrugated cardboard [10]. Sönmez and Özden’s research, meanwhile, concentrated on the effect of pigment coatings with different pigment ratios on dot gain during offset printing [11]. As demonstrated in the literature, current research on dot gain covers a variety of printing methods and substrates. Despite the significant progress having been achieved in understanding the phenomenon of dot gain, the urgent need remains to develop effective methods for controlling and correcting dot gain in the actual printing process [12].

The primary factors influencing dot gain encompass paper properties (e.g., smoothness, absorbency), ink properties (e.g., viscosity, drying speed), printing pressure, the number of screening lines, plate properties (e.g., hardness, flatness), and environmental conditions (e.g., temperature, humidity) [13–17]. These factors interact with each other, resulting in the degree of dot gain and different forms of expression. In actual printing, the complexity of the dot gain phenomenon is affected by a combination of factors. Traditional dot gain compensation methods, such as the direct subtraction method and the inverse function method, although theoretically feasible, suffer from insufficient accuracy or difficulty in practical application [8,18,19]. Consequently, the development of an efficient and accurate dot gain compensation strategy is imperative for enhancing printing quality and reducing production costs.

The application of mathematical modeling has emerged as a powerful approach for addressing complex industrial challenges. Recent studies across various fields have demonstrated the effectiveness of mathematical frameworks in simulating physical processes [20], developing predictive algorithms [21], establishing computational frameworks [22], and enhancing methodological rigor [23]. In printing research, mathematical modeling provides a robust methodology for tackling dot gain challenges by enabling quantitative characterization of this nonlinear phenomenon, decoupling complex factor interactions, and transforming empirical observations into predictive compensation strategies.

The innovative aspects of this study are mainly reflected in the following areas: First, a novel coordinate transformation-based compensation algorithm is proposed that effectively avoids the computational complexity of traditional inverse function methods while maintaining high precision. Second, a systematic experimental framework is established to comparatively analyze dot gain characteristics and compensation effects across three representative paper types (newsprint, offset paper, and coated paper) under different screen frequency (133 lpi and 175 lpi), providing comprehensive experimental data support. Furthermore, this research combines mathematical modeling with practical printing applications, developing a MATLAB-based compensation curve generation tool that offers printing enterprises a practical and operable technical solution.

In this study, the focus is on the utilization of the least squares method and MATLAB tools in conjunction with newsprint, offset paper, and coated paper as the research objects. The objective is to employ the “coordinate transformation method” to determine the dot compensation value and to establish a corrective compensation curve for dot gain. The efficacy of this approach is twofold: firstly, it enhances the accuracy and stability of lithographic printing, and secondly, it provides novel concepts and technical support for enhancing the quality of printing. It is anticipated that this method will provide an efficient and accurate dot gain compensation strategy for the printing industry and promote the further development of printing technology.

## 2 Related work

### 2.1 Compensatory methodology for dot gain

In the domain of printing, conventional methodologies for dot gain compensation primarily encompass the direct subtraction method and the inverse function method [24]. The direct subtraction approach entails the direct subtraction of the dot gain value, constituting a straightforward technique but exhibiting limited precision and substantial error [10,25]. The inverse function method, on the other hand, has been shown to achieve more accurate results by reversing the dot gain process. However, this method is computationally complex, particularly when polynomial fitting is involved, which can be challenging to solve and implement [8,26].

In recent years, there has been an increasing interest in compensation methods, with several studies exploring novel approaches. One such method involves the coordinate transformation technique, which circumvents the complexity of inverse function solving by altering the coordinate system, thus offering a more straightforward and pragmatic solution to compensate for dot gain [8]. Additionally, machine learning-based methods have been proposed to model the nonlinear relationship between dot gain and printing parameters. Nevertheless, the majority of these methods are primarily focused on theoretical derivation and simple experimental validation, with limited attention to comprehensive consideration of multiple printing conditions [27,28].

### 2.2 Mathematical modeling and curve fitting

The Least Squares Method (LSM) is a mathematical optimization technique that aims to find the best function match for data by minimizing the sum of squares of the errors [29]. It has a wide range of applications in statistics, signal processing, engineering, and data analysis. In the field of printing, the Least Squares Method is employed to fit dot gain curves, thereby identifying the optimal model parameters that minimize the sum of squared errors [8,30]. Polynomial fitting is a commonly used method for fitting dot gain curves, where a suitable polynomial order is selected [31]. However, high-order polynomials may lead to overfitting, especially when the data set is noisy or the sample size is limited [32].

To address the aforementioned challenges, several alternative fitting methods have been proposed. One such method is the segmented polynomial fitting method, which divides the curve into multiple parts, with each part being fitted with a simpler function. This approach has improved accuracy while maintaining computational efficiency [33]. Additionally, nonlinear fitting techniques, such as exponential or logarithmic models, have been employed to more accurately capture the nonlinear nature of dot gain [34]. When selecting a fitting method, it is imperative to consider data characteristics, model complexity, and practical application requirements to ensure optimal outcomes.

### 2.3 Printing processes and material properties

The printing process and material properties have been demonstrated to exert a substantial influence on dot gain. Studies have indicated that the surface properties of paper (e.g., smoothness, absorbency) and the physical properties of ink (e.g., viscosity, drying speed) function as the predominant factors affecting dot gain [35,36]. Furthermore, printing pressure, number of screening lines, and plate characteristics have been shown to significantly impact the extent of dot gain [37,38]. The interplay among these factors contributes to determining the final performance of the dot in the printing process.

In recent years, experimental analyses of dot gain patterns have been conducted under varying printing materials and conditions. For instance, Tang examined the differences in dot gain between offset printing paper, white board, and coated paper under identical printing conditions [39]. SESLİ, HAYTA, AKGÜL, and OKTAV conducted a comparative study of dot gain on coated and uncoated paper [40]. Conversely, Hao, Xu, and Chen investigated the impact of flexographic rubber and photopolymer plates on dot gain [41]. These studies provide a significant theoretical foundation for comprehending the phenomenon of dot gain. However, it remains challenging to synthesize the effects of multiple factors in practical applications.

In summary, although significant advancements have been made in dot gain compensation methods, mathematical modeling, printing processes, and material properties, the effective control and correction of dot gain in the actual printing process remains an urgent and unresolved issue. This study aims to propose an efficient and accurate dot gain compensation strategy. This strategy will be developed by integrating the least squares method and the coordinate transformation method, combined with a variety of printing conditions and material properties. This strategy is expected to provide new ideas and technical support for quality improvement in the printing industry.

## 3 Method

All numerical methodologies described in this section (including the coordinate transformation algorithm and least-squares fitting) were applied to a robust empirical dataset of 120 measurements, encompassing three paper types under two screen frequency, as detailed in Section 3.4.1.

### 3.1 Algorithm for dot gain compensation

Dot gain compensation represents a fundamental challenge in printing quality control, traditionally addressed through various mathematical approaches [42,43]. While conventional methods such as the direct subtraction method and inverse function method have been widely employed, they present significant limitations in practical applications. The direct subtraction method, though computationally simple, often lacks precision due to its failure to account for the nonlinear nature of dot gain [8]. Conversely, the inverse function method, while theoretically sound, introduces substantial computational complexity, particularly when dealing with higher-order polynomial fitting, making it less suitable for industrial applications where processing efficiency is crucial [18,19].

**Fig 1:**
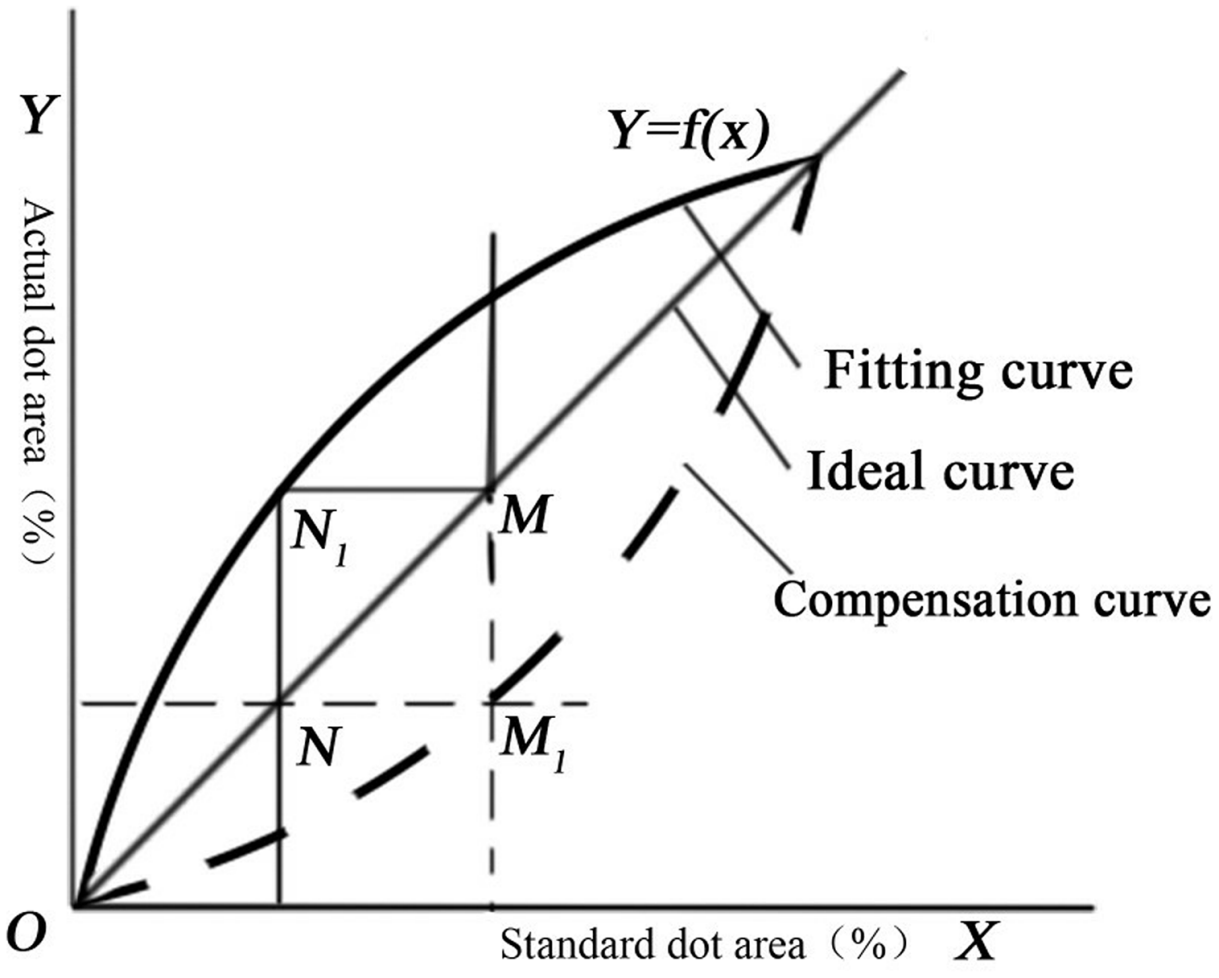
The coordinate transformation method for dot gain compensation. This approach transforms the compensation problem into a geometric mapping exercise, avoiding the mathematical complexity associated with traditional inverse function solutions while maintaining comparable accuracy.

To deepen the understanding of system dynamics and address these limitations, this study adopts the “coordinate transformation method” (Fig 1), which offers a novel approach to dot gain compensation. The theoretical foundation of this methodology lies in its ability to transform the compensation problem into a coordinate mapping exercise, thereby avoiding the mathematical complexity associated with traditional inverse function solutions while maintaining comparable accuracy [8]. This approach was selected not only for its effectiveness in handling nonlinear relationships in printing processes but also for its ability to intuitively reveal the intrinsic dynamics of the dot gain system through geometric transformation, while remaining computationally tractable for practical implementation.

The implementation framework comprised four systematic steps: First, a mathematical model of dot gain was constructed using polynomial fitting techniques to establish the fitted curve. Second, a vertical projection was extended from point M to intersect the fitted curve at point N₁. Subsequently, a horizontal projection from N₁ establishes intersection point N with the ideal curve. Finally, the compensation point M₁ was derived through the intersection of vertical and horizontal projections from points M and N, respectively. This methodological approach effectively transformed the compensation challenge into a geometric coordinate transformation problem, significantly reducing computational overhead while enhancing the understanding of system dynamics in dot gain compensation through intuitive geometric relationships.

The advantages of this methodology are threefold: First, it eliminates the need for complex inverse function calculations, thereby enhancing computational efficiency; Second, it maintains high precision in compensation curve generation through systematic coordinate mapping; and Third, it provides an intuitive graphical representation that facilitates both industrial implementation and the understanding of dynamic relationships between system parameters. However, it should be noted that this method’s performance is highly dependent on the accuracy of the initial curve fitting process. Furthermore, its application to novel printing conditions or unconventional materials may require additional validation and potential algorithmic adjustments, presenting avenues for future research while revealing the sensitivity of system dynamic behavior to initial conditions.

### 3.2 Least squares-based curve fitting

The Least Squares Method (LSM) was selected as the optimization framework for this study due to its well-established theoretical foundation, proven effectiveness in handling empirical data with measurement uncertainties, and its capability to reveal intrinsic system dynamics through parameter estimation [44–46].The fundamental principle of LSM involves minimizing the sum of squared errors between observed data and model predictions, expressed mathematically as:


$$S=\sum {\mathrm{\backslash nolimits}}_{i=\text{1}}^{n}(y_{i}{-f(x_{i}))}^{\text{2}}$$ (1)

where *S* is the sum of squared errors; *n* is the number of observed data points; $y_{i}$is the actual output value of the *i*-th observed data point; and $f(x_{i})$is the model-predicted output value based on the input value $x_{i}$.

The choice of polynomial fitting for dot gain curve modeling was based on multiple considerations, primarily because this approach effectively captures system dynamic behavior: First, the complex nonlinear relationship between nominal dot area and actual dot gain necessitates a flexible mathematical framework capable of capturing various curve shapes [47]. Second, polynomial functions offer excellent adaptability to empirical data patterns while maintaining mathematical tractability [48]. After comprehensive evaluation of multiple function types and orders, a third-order polynomial was selected as it provides the optimal balance between model complexity and fitting accuracy while effectively characterizing the nonlinear dynamics of the dot gain system:


$$y_{n-\text{1}}=C_{\text{0}}+C_{\text{1}}X+C_{\text{2}}X^{\text{2}}+C_{\text{3}}X^{\text{3}}$$ (2)

where $y_{n-\text{1}}$ is the output value of the fitted curve, $C_{\text{0}}$, $C_{\text{1}}$, $C_{\text{2}}$, $\;C_{\text{3}}$ are the polynomial coefficients, and *X* is the input value.

This specific polynomial order was determined through iterative testing and residual analysis, ensuring sufficient flexibility to capture the characteristic S-shape of dot gain curves while avoiding overfitting issues associated with higher-order polynomials. The implementation through MATLAB’s polyfit function further ensured computational efficiency and numerical stability, which is crucial for processing substantial experimental data and guaranteeing the reliability of system dynamics analysis.

### 3.3 Ethical approval

This study involved mathematical modeling and analysis of color measurement data obtained from standard printed materials. As the research did not involve human participants, animal subjects, or any sensitive cultural artifacts, it did not require review or approval from an ethics committee or other governmental bodies.

### 3.4 Inclusivity in global research

Additional information regarding the ethical, cultural, and scientific considerations specific to inclusivity in global research is included in the Supporting Information (S1 File).

A detailed, step-by-step protocol for the measurement and compensation of dot gain is available as Supporting Information (S1 Text).

## 4 Experiments

### 4.1 Experimental materials and equipment

#### 4.1.1 Software.

CorelDraw 2019 (used for test sheet design).

MATLAB 9.0 (R2016a) (used for data analysis and curve fitting).

#### 4.1.2 Materials.

**Ink:** Tianjin Dongyang TGO-NO four-color non-skinning offset ink (viscosity: 8–13 (S), setting speed ≤ 15 min, drying time ≤ 20–40 min, fluidity ≥ 30–36 mm).

**Paper:**

Shandong Chenming newsprint (48 g/m²).

Shandong Chenming offset paper (120 g/m²).

Shandong Chenming coated paper (150 g/m²).

#### 4.1.3 Equipment.

**Printer:** Jiangsu Zhongjing PZ4740E-AL four-color offset press (maximum printing area: 540 × 740 mm, maximum printing speed: 12,000 sheets/hour, paper thickness range: 0.04–0.60 mm).

**Spectrophotometer:** X-Rite eXact spectrophotometer (D50 light source, 2° observation angle, density repeatability: ± 0.01).

### 4.2 Experimental conditions

(a) Testing environment:
  **Temperature:** 23–28°C.
  **Relative humidity:** 55%−65%
(b) Print order: K (Black) → M (Magenta) → C (Cyan) → Y (Yellow)
(c) Dot shape: Circular dots
(d) Screen line number: 133 lines per inch (lpi), 175 lines per inch (lpi)

### 4.3 Design test samples

**Fig 2:**
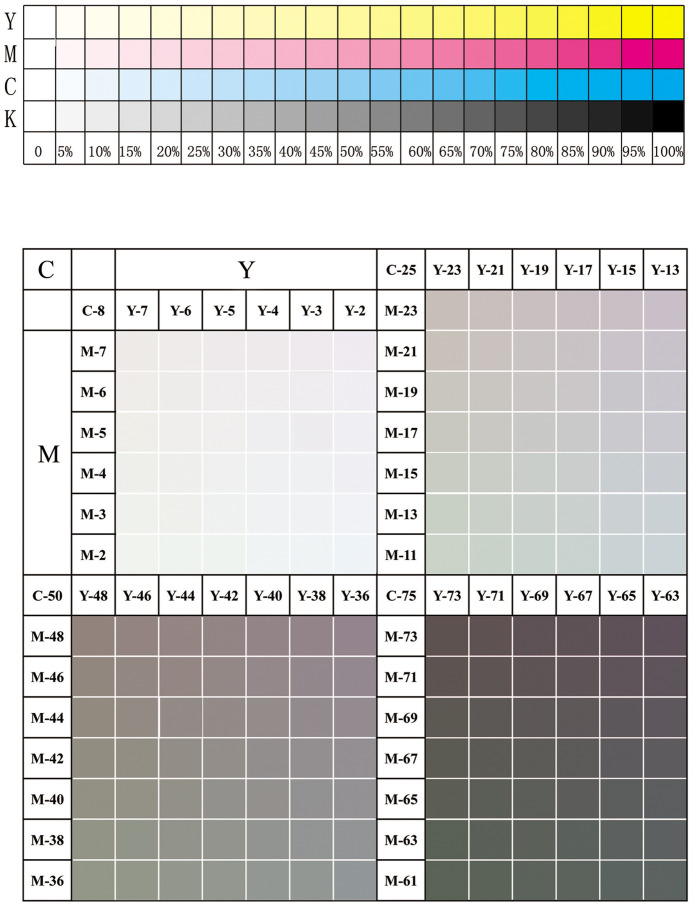
Samples of dot gain tests. The layout includes a gray balance test section with C/M/Y patches at four tone levels (8%, 25%, 50%, 75%), and a gradient section with four-color (C, M, Y, K) scales from 0% to 100% for dot gain measurement.

Using CorelDraw 2019 software, test samples were designed as shown in Fig 2. The test samples include a gray balance test area and a gradient test area.

**Grey balance test area:** This area evaluates gray balance at four tonal levels: 8%, 25%, 50%, and 75%. It consists of a series of color patches formed by combinations of cyan (C), magenta (M), and yellow (Y).

**Gradient test area:** This area contains four-color (C, M, Y, K) scales, each ranging from 0% to 100% in increments. These scales are used to measure dot gain.

### 4.4 Experimental procedures

**Fig 3:**
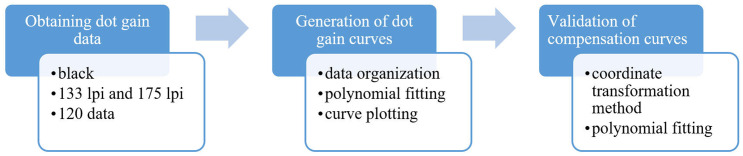
Experimental procedures. The procedure involves three main steps: (1) acquiring dot gain data using black ink at 133/175 lpi screen frequency; (2) generating dot gain curves through data organization and polynomial fitting; (3) validating compensation curves via coordinate transformation and polynomial fitting.

The experimental procedure is shown in Fig 3 and consists of the following main steps.

#### 4.4.1 Obtaining dot gain data.

Dot gain data for three types of paper (newsprint, offset, coated) were measured at two different screen frequency (133 lpi and 175 lpi) for black (K) using an X-Rite eXact spectrophotometer. Each measurement was repeated three times for every dot percentage (from 5% to 100% in 5% increments) to ensure reliability, and the average values were used for subsequent numerical analysis. This rigorous approach yielded a comprehensive dataset of 120 data points, derived from 3 paper types, 2 screen frequency, and 20 dot areas (S1 Data), which served as the fundamental input for all subsequent numerical simulations and model fitting in this study.

#### 4.4.2 Dot gain curve generation.

**Data organization:** MATLAB software was used to import and organize the 120 collected data points.

**Polynomial fitting:** Using the principle of least squares, the polyfit function in MATLAB was used to fit a third-degree polynomial to the data under two screen frequency, 133 lpi and 175 lpi.

**Plotting curves:** The polyval function was used to calculate the fitting polynomial in the specified range of function values, and the plot function was used to draw the dot gain curve. By comparing different types of paper and the net line count of the curve, you can analyze the pattern of dot gain change.

The following is the Matlab code for obtaining and fitting the dot gain curve for coated paper:

*% Data*

dot_area = 5:5:100;

coatedpaper_133lpi = [2 4 8 9 9 10 11 10 11 12 13 13 17 16 18 13 8 7 5 0];

coatedpaper_175 lpi = [3 6 8 10 13 14 17 17 18 19 20 21 23 23 20 17 11 8 5 0];

*% Polynomial fitting*

p_ coatedpaper_133lpi = polyfit(dot_area, coatedpaper_133lpi, 3);

p_ coatedpaper_175lpi = polyfit(dot_area, coatedpaper_175lpi, 3);

*% Generate fitting curves*

fitted_dot_area = linspace(5, 100, 100);

fitted_coatedpaper_133lpi = polyval (p_coatedpaper_133lpi, fitted_dot_area);

fitted_coatedpaper_175lpi = polyval (p_coatedpaper_175lpi, fitted_dot_area);

*% plotting the fitting curve*

plot(fitted_dot_area, fitted_coatedpaper_133lpi, ‘-’, ‘LineWidth’, 1, ‘Color’, ‘b’, ‘DisplayName’, ‘Fitting Curve(133lpi)’);

plot(fitted_dot_area, fitted_coatedpaper_175lpi, ‘-’, ‘LineWidth’, 1, ‘Color’, ‘r’, ‘DisplayName’, ‘Fitting Curve(175lpi)’);

#### 4.4.3 Calculation and validation of dot gain compensation curves.

Using the “coordinate transformation method”, the horizontal and vertical coordinates of the original data points were swapped, and the dot gain compensation curves were inversely fitted under two screen line number conditions. The polynomial fitting equation for the compensation curve was obtained using the Poly2str function of MATLAB. Furthermore, the plot function was used to plot the dot gain compensation curve. Finally, the calculated compensation curves were applied to actual printing tasks. The effectiveness of the compensation was validated using a comprehensive dataset of 450 measured dot area values covering three paper types and two screen frequency (S2 Data).The dot gain and improvement in printing effect after compensation were evaluated by comparing the dot output curves before and after compensation with the ideal printing curves.

## 5 Results

### 5.1 Polynomial fitting of dot gain curves and dot gain analysis

The polynomial fitting and subsequent analysis presented in this section are based entirely on the experimental dot gain data detailed in Section 3.4.1. All numerical simulations, including curve fitting and compensation calculations, were performed using this dataset.

#### 5.1.1 Dot gain analysis for three types of paper.

**Fig 4:**
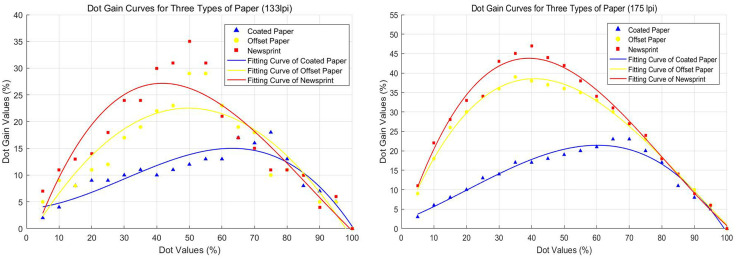
Dot gain curves for three types of paper at different screen frequency. (a) 133 lpi and (b) 175 lpi. Dot gain initially increases and then decreases with rising dot area percentage, showing the most significant expansion in the mid-tone range (approximately 40%–60%) across coated, offset, and newsprint papers.

Figs 4a and 4b show the dot gain curves of coated paper, offset paper, and newsprint at 133 lpi and 175 lpi respectively. From the overall trend, it can be seen that as the dot value increased, the dot gain of the three types of paper first increased and then decreased. This indicated. This indicated that in the printing process, the actual size of the dot value changed with the dot value and change, and in the mid-tone (dot value of about 40% − 60%), the phenomenon of dot expansion was most significant.

The dot gain of the three types of paper varied significantly at different screen frequency. The dot gain value for coated paper was relatively small and the curve was relatively flat. Below 133 lpi the dot gain was around 15%, below 175 lpi the dot gain ranged from 15% − 25%. Dot gain values for offset paper were in the middle range, with a steeper curve. At 133 lpi the dot gain was around 25%; at 175 lpi the dot gain ranged between 25% and 35%. Newsprint had the highest dot gain and the steepest curve. At 133 lpi the dot gain was around 30%; at 175 lpi the dot gain ranged between 35% and 45%.

#### 5.1.2 Analysis of dot gain for different screen frequency.

**Fig 5:**
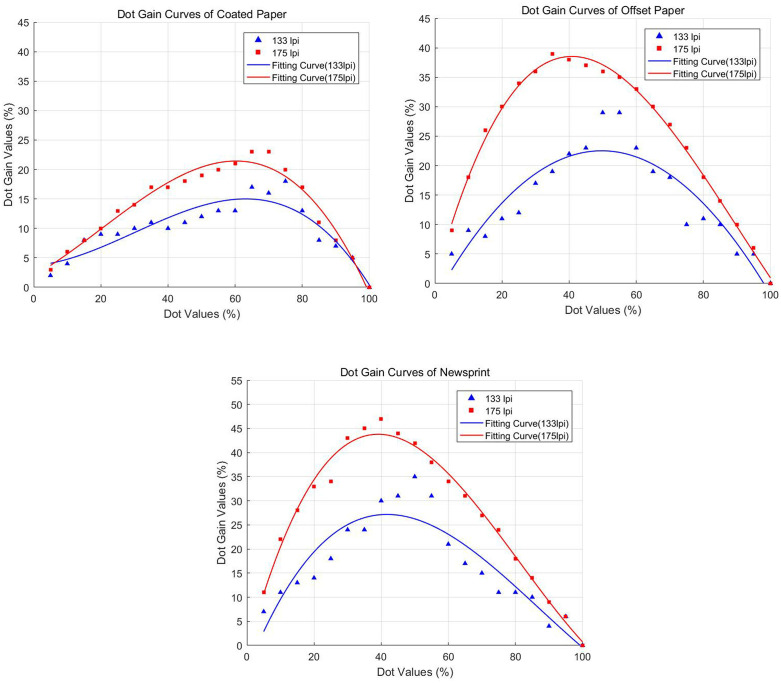
Comparison of dot gain across paper types and screen frequency. (a) Coated paper. (b) Offset paper. (c) Newsprint. Dot gain curves for 133 lpi and 175 lpi show a consistent non-linear trend, rising to a maximum in the mid-tone range (40%–60% dot area) before decreasing. Mid-tone regions exhibited the most significant dot gain across all paper types.

Figs 5a-5c show the dot gain curves for coated paper, offset paper, and newsprint at screen frequency of 133lpi and 175lpi respectively. Overall, the dot gain values showed an increasing and then decreasing trend as the dot count increases. This is a common phenomenon in the printing process, i.e., in the mid-tone area (dot values around 40% − 60%) the dot gain is most obvious. This showed that in printing, the actual size of the dot changes with the dot value, and the mid-tone area is more affected by dot gain.

For coated paper, dot gain values at 133 lpi ranged from approximately 5% to 20%, while at 175 lpi they ranged from approximately 10% to 30%. For offset paper, dot gain values at 133 lpi ranged from approximately 10% to 25%, while at 175 lpi they ranged from approximately 15% to 35%. For newsprint, dot gain values at 133 lpi ranged from approximately 10% to 30%, while at 175 lpi they ranged from approximately 20% to 50%.

### 5.2 Compensation and validation of dot gain

#### 5.2.1 Dot gain compensation.

**Fig 6:**
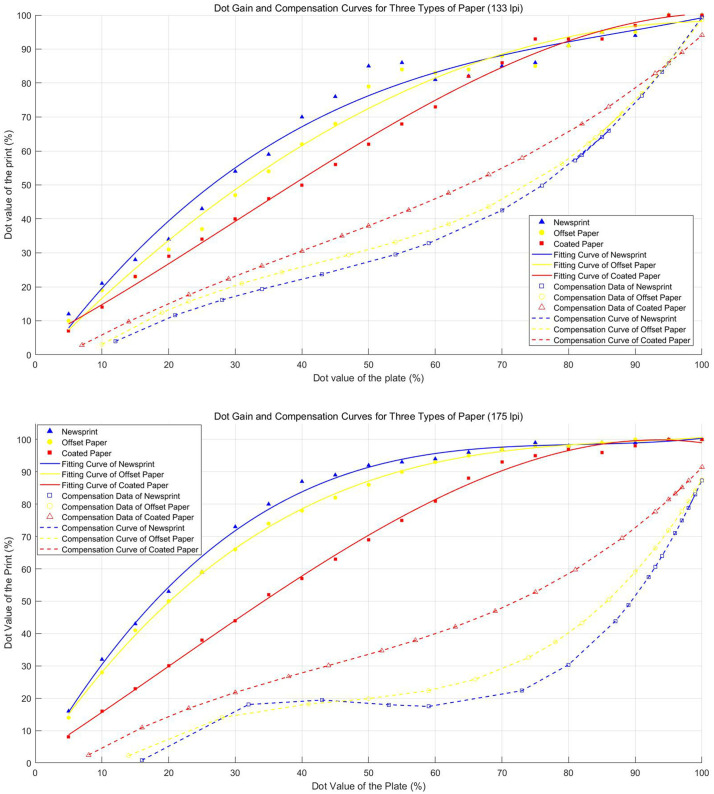
Dot fitting &compensation curve. (a) 133 lpi. (b) 175 lpi. Curves were derived via MATLAB for coated, offset, and newsprint papers. The coordinate transformation method effectively generated compensation curves to counteract nonlinear dot gain.

In this study, the “coordinate transformation method” was used to generate the compensation curve for dot gain. MATLAB software was used to convert the data and obtain the equations of the fitted curves and compensation curves. Table 1 shows the equations of the dot fitting and compensation curves for three types of paper under the screen frequency of 133 lpi and 175 lpi. Fig 6a and 6b illustrate the dot fitting and compensation curves for three types of paper under these two screen frequency, respectively.

**Table 1:** Equations for dot fitting and compensation curves.

Screen Frequency	Plate	Fitting Curve Equations	Compensation Curve Equation
**175lpi**	Coated Paper	$\text{y}=-\text{8}.\text{8602e}-\text{05}*{\text{x}}^{\text{3}}+\text{0}.\text{0053}*{\text{x}}^{\text{2}}\;+\text{1}.\text{3243}*\text{x}+\;\text{1}.\text{9676}$	$\text{y}=-\text{1}.\text{6384e}-\text{04}*\;{\text{x}}^{\text{3}}-\text{0}.\text{0214}*{\text{x}}^{\text{2}}+\;\text{1}.\text{4932}*\text{x}=-\;\text{8}.\text{1872}$
	Offset Paper	$\text{y}=\text{1}.\text{1845e}-\text{04}*\;{\text{x}}^{\text{3}}-\text{0}.\text{0324}*{\text{x}}^{\text{2}}+\text{3}.\text{0530}*\text{x}+\text{0}.\text{6384}$	$\text{y}=\text{3}.\text{6073e}-\text{04}*{\text{x}}^{\text{3}}-\text{0}.\text{0491}*{\text{x}}^{\text{2}}+\text{2}.\text{4076}*\text{x}-\text{22}.\text{7929}$
	Newsprint	$\text{y}=\text{1}.\text{7536e}-\text{04}*{\text{x}}^{\text{3}}-\text{0}.\text{0430}*{\text{x}}^{\text{2}}+\text{3}.\text{5599}*\text{x}-\text{1}.\text{0312}$	$\text{y}=\text{6}.\text{0695e}-\text{04}*{\text{x}}^{\text{3}}-\text{0}.\text{0905}*{\text{x}}^{\text{2}}+\text{4}.\text{3337}*\text{x}-\text{47}.\text{8140}$
**133lpi**	Coated Paper	$\text{y}=-\text{7}.\text{9166e}-\text{05}*{\text{x}}^{\text{3}}+\text{0}.\text{0072}*{\text{x}}^{\text{2}}+\text{1}.\text{0430}*\text{x}+\text{3}.\text{6927}$	$\text{y}=\text{9}.\text{3206e}-\text{05}*{\text{x}}^{\text{3}}-\text{0}.\text{0114}*{\text{x}}^{\text{2}}+\text{1}.\text{1931}*\text{x}-\text{4}.\text{9467}$
	Offset Paper	$\text{y}=\text{5}.\text{0199e}-\text{06}*{\text{x}}^{\text{3}}-\text{0}.\text{0106}*{\text{x}}^{\text{2}}+\text{2}.\text{0210}*\text{x}-\text{2}.\text{5501}$	$\text{y}=\text{2}.\text{2658e}-\text{04}*{\text{x}}^{\text{3}}-\text{0}.\text{0290}*\;{\text{x}}^{\text{2}}+\text{1}.\text{7322}*\text{x}-\text{11}.\text{6044}$
	Newsprint	$\text{y}=\text{1}.\text{0242e}-\text{04}*{\text{x}}^{\text{3}}-\text{0}.\text{0270}*{\text{x}}^{\text{2}}+\text{2}.\text{7231}*\text{x}-\text{5}.\text{0687}$	$\text{y}=\text{2}.\text{2615e}-\text{04}*{\text{x}}^{\text{3}}-\text{0}.\text{0272}*{\text{x}}^{\text{2}}\;+\text{1}.\text{5650}*\text{x}-\text{11}.\text{3151}$

Screen Frequency: The resolution of the halftone screen in lines per inch (lpi).

Plate: The type of printing plate used in the experiment.

Fitting Curve Equation: The polynomial equation (cubic fit) modeling the dot gain behavior.

Compensation Curve Equation: The polynomial equation (cubic fit) used to compensate for dot gain.

#### 5.2.2 Validation of dot gain compensation.

**Fig 7:**
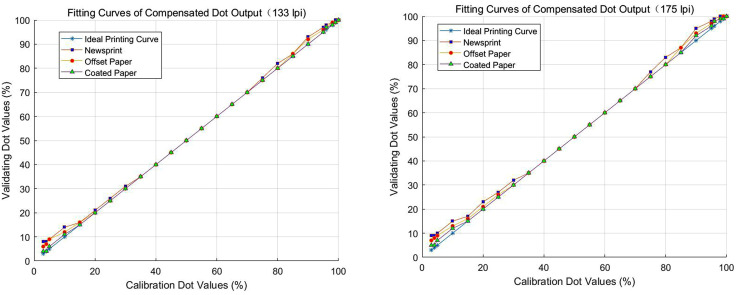
Fitting curves of compensated dot output versus ideal printing curves. (a) 133 lpi. (b) 175 lpi. Compensated dot output curves for newsprint, offset paper, and coated paper closely align with the ideal printing curve, demonstrating the effectiveness of the dot gain compensation method across different paper types and screen frequency.

To evaluate the actual effect of the dot gain compensation curve, this study analyzed the comparison of two screen frequency: 133 lpi and 175 lpi. The results of the study are shown in Fig 7a and 7b, where the fitted curves of the compensated dot output of newsprint, offset paper, and coated paper show a similar trend in comparison with the ideal printing curves for the three types of paper under both screen frequency.

At low dot values (0–20%) in the bright tone area, the dot output curves for all three types of papers deviated somewhat from the ideal printing curve for both the 133lpi and 175lpi. This result shows that in the bright tone area, the dot gain compensation effect still had room for further improvement. However, in the mid-tone (20% − 80%) area, the dot gain curves of the three papers are gradually approached the ideal printing curve at both screen frequency, indicating that the dot gain compensation effect in the mid-tone area was better and could achieve the ideal reproduction effect. In addition, in the high dot value (80% − 100%) of the dark-tone area, although there was a slight deviation between the two screen frequency, the overall dot output curve was closer to the ideal printing curve, indicating that the compensation effect of the dark-tone area was also more satisfactory.

In summary, the dot gain compensation effect under the two screen frequency performed well in the mid-tone and dark-tone areas, and the dot output curve was closer to the ideal printing curve, which indicated that the compensation measures could effectively improve the dot reproduction situation in these areas. However, in the bright tone area, the dot output curve deviated from the ideal printing curve to a more obvious extent, indicating that the compensation effect in the bright tone area still needs to be further optimized. Although there were slight differences in the degree of proximity between the dot gain compensation curves and the ideal printing curves for the three types of paper under different screen frequency, the overall dot gain compensation measures improved the dot replication to a certain extent, making the printing effect closer to the ideal state. This result provides an important reference for the subsequent optimization of the dot gain compensation curve but also provides theoretical support for the improvement of the printing process.

## 6 Discussion

### 6.1 Analysis of dot gain phenomenon for three types of paper

The experimental results showed a significant difference in the dot gain phenomenon among coated paper, offset paper, and newsprint. The relatively small value of dot gain for coated paper was mainly due to its extremely smooth surface and very low ink diffusion [49], which can help to maintain the shape and size of the dot better [50]. This characteristic helps the coated paper to present high-quality printing results at both screen frequency, which is especially suitable for fine image printing. In contrast, newsprint had the most significant dot gain, with a rough surface that is highly susceptible to ink penetration and diffusion, resulting in blurred dot edges [51] and high dot gain values. This characteristic makes the newsprint in the printing of the dot area change, the stability of the printing effect is poor, but because of its low cost, it is commonly used in newspaper printing where quality requirements are relatively not high. Offset paper dot gain value was in the middle range, its surface has a certain degree of roughness, and the degree of ink diffusion is between coated paper and newsprint [52,53]. Under reasonable printing conditions, offset paper can still help to control ink diffusion better [54], but the dot area change is relatively large, making it suitable for general printing needs.

### 6.2 Analysis of dot gain for different screen frequency

Different screen frequency also had a significant effect on dot gain. 175 lpi had a higher dot gain than 133 lpi, which was mainly because 175 lpi has a denser distribution of dots with smaller dot spacing [55], which makes it easier for ink to diffuse and penetrate into neighboring dots during printing, resulting in blurring of the edges of the dots [56], and the phenomenon of dot gain is more obvious. In contrast, the dot spacing of 133 lpi is relatively large, and the effect of ink diffusion on dot gain is relatively small, so the degree of dot gain is relatively mild. This result shows that the selection of screen line count has an important impact on the printing quality, which needs to be selected reasonably according to the specific printing requirements and paper characteristics.

### 6.3 Analysis of the compensation effect of dot gains

In this study, the dot gain compensation curve generated by the “coordinate transformation method” improved the dot printing quality to a certain extent. In terms of the compensation effect, the dot output curves in the mid-tone and dark-tone areas were closer to the ideal printing curves, indicating that the compensation measures in these areas can effectively improve the dot replication and make the printing effect closer to the ideal state. This successful compensation, facilitated by the least-squares-fitted model, quantitatively reveals a key system dynamic: the nonlinear relationship between nominal and actual dot area is predominantly consistent and predictable across mid and dark tones.

However, in the bright tone area (0–20%), the dot output curve deviated from the ideal printing curve, which may be related to the fact that the dot in the bright tone area is smaller, and the effect of ink diffusion on the dot shape is more significant. Crucially, this specific failure of the polynomial model is itself highly informative, exposing a fundamental shift in the system dynamics within the highlight region. The physical processes here (e.g., ink trapping, dot sharpness on substrate) likely operate under a different regime that cannot be captured by a single global model. Thus, the model not only provides a tool for compensation but also serves as a diagnostic probe for identifying complex dynamic behaviors within the printing system.

Future research can further optimize the compensation method in the bright tone area, for example, by adjusting the parameters of the compensation curve or adopting a more complex compensation model to improve the compensation accuracy.

### 6.4 Limitations of the study and prospects

Although this study has achieved some results in dot gain compensation, there are still some limitations. For example, the experiments involved only three common paper types, and the applicability to other special papers has not been verified. In addition, the generation of the compensation curves was based on polynomial fitting, which may not be able to fully capture the complex nonlinear characteristics of dot gain [48]. Future research could consider introducing more advanced fitting methods, such as machine learning algorithms, to improve the accuracy and adaptability of the compensation curves. Meanwhile, further studies on the effects of different printing conditions (e.g., ink type, printing pressure, etc.) on dot gain compensation will help to refine the compensation strategy and make it applicable in a wider range of printing scenarios.

## 7 Conclusion

In this study, the dot gain data of three types of paper (newsprint, offset paper, coated paper) under different screen frequency (133 lpi, 175lpi) were collected by designing a dot gain printing test plate, and the data processing and compensation strategy were investigated based on the least squares method using MATLAB software. The main innovations of this research lay in the development of a novel coordinate transformation-based compensation algorithm that avoids the computational complexity of traditional inverse function methods, and the establishment of a systematic experimental framework for comparative analysis across multiple paper types and screen frequency. It was found that the dot gain phenomenon in different paper and screen frequency showed significant differences: coated paper had the smallest dot gain value with a flat curve, offset paper was in the middle range, newsprint had the largest dot gain with a steep curve, which is mainly related to the surface characteristics of the paper and the degree of ink diffusion. As the screen frequency increased, the dot gain phenomenon became more obvious.

More significantly, the employed methodology transcends its practical utility by providing profound insights into the system dynamics of the dot gain phenomenon. The integration of the Least Squares method and the coordinate transformation framework serves as a powerful diagnostic probe, quantitatively revealing that the nonlinear dynamics of dot gain are consistent and predictable in mid-to-dark tones, while exposing a fundamental shift in the underlying physical mechanisms within the highlight region. This enhanced mechanistic understanding moves beyond empirical observation towards a model-based comprehension of the system’s behavior.

In this study, the compensation curve was generated by the “coordinate transformation method”, which effectively improved the reproduction effect of printing dots. However, the compensation effect still needs to be optimized in the bright tone area. This study provides a least squares-based dot gain compensation strategy for the printing industry, which can effectively improve printing accuracy and stability. Through experimental verification, this strategy showed a good compensation effect in the mid-tone and dark-tone areas, demonstrating the practical value of the proposed innovative methodology. The research provides new ideas and technical support for the improvement of printing quality.

Future research will focus on further optimizing the existing dot gain compensation algorithm to develop a strategy more suitable for the bright tone area. Additionally, the influence of factors such as different inks and printing equipment on dot gain and compensation effectiveness will be investigated to promote continuous improvement in the printing process.

## Supporting information

S1 File Questionnaire. Inclusivity in global research questionnaire. The completed questionnaire regarding ethical, cultural, and scientific considerations specific to inclusivity in global research, as required by PLOS ONE.
(DOCX)

S1 Data Data on dot gain for three types of paper. This file contains the complete dataset of color measurements used to construct and fit the dot gain compensation model. Data includes measurements from three paper types under two screen frequency.
(XLSX)

S2 Data Data on validation of compensation curves for dot gain. This file contains the independent dataset of measurements used exclusively for validating the predictive accuracy and effectiveness of the fitted compensation equation.
(XLSX)

S1 Text Protocol. Experimental protocol for dot gain measurement and modeling. This file details the standardized procedures for measuring dot gain, mathematical modeling, and generating compensation curves. The protocol covers sample preparation, instrumental measurement, data fitting, and model validation to ensure experimental reproducibility and result reliability.
(DOCX)
