# Supplementary material for: Mathematical modelling and compensation strategies for printing dot gain
Source: PLoS One. 2025 Oct 30;20(10):e0334921. doi: 10.1371/journal.pone.0334921 (PMC12574880; doi:10.1371/journal.pone.0334921)
Supplement: S1 Text — Experimental protocol for dot gain measurement and modeling. This file details the standardized procedures for measuring dot gain, mathematical modeling, and generating compensation curves. The protocol covers sample preparation, instrumental measurement, data fitting, and model validation to ensure experimental reproducibility and result reliability. (DOCX) [file pone.0334921.s004.docx]

**Measuring and Compensating for Dot Gain in Offset Printing Using Coordinate Transformation and Least Squares Fitting**

**Authors:** Xiaoli LIU^1^, Bingzhong QIU^1*^, Ying-Leh Ling^2^

**Affiliation:**

^1^ General Education Department, Qilu Medical University, Shandong, China

^2^ Department of Mathematics, Science and Computer, Politeknik Kuching Sarawak, Sarawak, Malaysia

**Keywords:** least squares method, coordinate transformation method, dot gain, compensation curve, screen line count

**1. Introduction**

This detailed protocol describes a systematic approach for measuring, modeling, and compensating for dot gain (Tone Value Increase, TVI) in offset printing. The core of this method involves applying the "coordinate transformation method" combined with least-squares polynomial fitting to generate high-precision dot gain compensation curves, thereby effectively improving print quality. This protocol is an integral part of the submitted research paper, "*Mathematical modelling and compensation strategies for printing dot gain*," and provides complete, reproducible, and detailed steps for the operations described in the "Experiments" section (Section 3) of the paper, ensuring transparency and reproducibility of the research.

**2. Materials**

**2.1 Software**

CorelDraw 2019, MATLAB R2016a

**2.2 Consumables**

**Ink:** Tianjin Dongyang TGO-NO four-color non-skinning offset ink (C, M, Y, K).

**Paper:**

Shandong Chenming Newsprint (48 g/m²)

Shandong Chenming Offset Paper (120 g/m²)

Shandong Chenming Coated Paper (150 g/m²)

**2.3 Equipment**

**Printer:** Jiangsu Zhongjing PZ4740E-AL four-color offset press (or similar model with precise calibration).

**Spectrophotometer:** X-Rite eXact (or similar model) calibrated to D50 light source and 2° standard observer.

**3. Procedure**

**3.1 Environmental Preparation**

The experiment shall be carried out under constant ambient conditions: temperature 23–28°C, relative humidity 55–65%.

**3.2 Design and Print the Test Target**

**3.2.1 Design the test form**

Using CorelDraw, create a document with the layout shown in Figure 2 of the main manuscript.

- **Gradient Test Area:** Create four sets of gradients (one for each process color C, M, Y, K). Each gradient should range from 0% to 100% dot area in 5% increments (e.g., 5%, 10%, 15%, ... 100%). Use circular dot shape.
- **Grey Balance Test Area:** Include patches for grey balance assessment at 8%, 25%, 50%, and 75% tone levels using combinations of C, M, and Y.

**3.2.2 Output the plates**

Output the digital file to plates for the offset press at the required screen frequencies: 133 lpi and 175 lpi.

**3.2.3 Print the test forms**

On the offset press, print the test forms on the three paper types.

- **Printing Order:** K → M → C → Y.
- Ensure the press is properly calibrated and ink-water balance is optimized before printing production sheets.
- Collect multiple sheets to ensure you have clean, representative samples for measurement.

**3.3 Measure Dot Gain Data**

**3.3.1 Calibrate the spectrophotometer**

Power on the X-Rite eXact and perform zero calibration according to the manufacturer's instructions.

**3.3.2 Measure dot gain**

- For each paper type and screen frequency, measure the printed dot area % for each step in the black (K) ink gradient. Measure each dot percentage three times and record the average value. This will minimize measurement error.
- **Data Structure:** You should end up with 6 datasets (3 paper types × 2 screen frequencies). Each dataset contains 20 data points (for dot areas 5% to 100% in 5% steps) of the measured (printed) dot area.

**3.4 Data Analysis and Curve Fitting in MATLAB**

**3.4.1 Organize data in MATLAB**

Create vectors in MATLAB for the nominal dot areas (input x) and the averaged measured dot gain values (output y).

***Example for Coated Paper, 175 lpi:***

% Input: Nominal Dot Area %

dot_area = 5:5:100;

% Output: Measured Dot Area % for Coated Paper, 175lpi

coatedpaper_175lpi_measured = [3 6 8 10 13 14 17 17 18 19 20 21 23 23 20 17 11 8 5 0];

**3.4.2 Perform polynomial fitting**

Use the polyfit function to fit a 3rd-order polynomial to the data.

***Example:***

% Fit a 3rd-order polynomial

p_coated_175lpi = polyfit(dot_area, coatedpaper_175lpi_measured, 3);

% Display the polynomial coefficients (C0, C1, C2, C3)

disp(p_coated_175lpi);

% Create a function for the fitted curve

fitted_dot_area = linspace(5, 100, 100); % Generate 100 points for a smooth curve

fitted_curve = polyval(p_coated_175lpi, fitted_dot_area);

**3.4.3 Plot the fitted dot gain curves**

Use the plot function to visualize the original data and the fitted curve for each paper/screen combination.

***Example:***

figure;

plot(dot_area, coatedpaper_175lpi_measured, 'o', 'DisplayName', 'Measured Data'); % Plot measured data points

hold on;

plot(fitted_dot_area, fitted_curve, '-r', 'LineWidth', 2, 'DisplayName', 'Fitted Curve (3rd order)'); % Plot fitted curve

xlabel('Nominal Dot Area (%)');

ylabel('Measured Dot Area (%)');

title('Dot Gain Curve - Coated Paper, 175 LPI');

legend('show');

grid on;

hold off;

**3.5 Generate the Compensation Curve**

**3.5.1 Apply the coordinate transformation**

The core of the method is to swap the X and Y coordinates of the fitted curve to generate the compensation curve. In practice, you will use the fitted polynomial to find the inverse relationship.

**3.5.2 Fit the compensation curve**

Generate a vector of desired output dot percentages (e.g., from 5% to 100%).For each desired output Y_desired, use the fitted polynomial and a solver (or simple coordinate swap on the fitted curve data points) to find the corresponding input X_comp. Fit a new 3rd-order polynomial to these (Y_desired, X_comp) data points. This new polynomial is your compensation curve equation.

**Note:** *This can be implemented programmatically using fsolve to invert the original fitted polynomial for each point, but the graphical coordinate swap method described in the paper (Fig. 1) is conceptually equivalent and simpler to implement for generating a set of points for a new fit.*

**3.5.3 Output the compensation equation**

Use the poly2str function to display the equation of the compensation curve in a readable format.

***Example:***

comp_eq_str = poly2str(p_compensation, 'x');

disp(['Compensation Curve Equation: y = ', comp_eq_str]);

**3.6 Validation of Compensation Effectiveness**

*(This step is crucial for demonstrating the practical value and accuracy of the proposed compensation strategy on an independent, more detailed dataset.)*

**3.6.1 Design a New, Detailed Validation Target**

This target should contain a more detailed gradient of nominal dot percentages (25 steps from 3% to 100%) compared to the initial target used for model building.Critically, use the compensation curve equations obtained in Section 3.5 to pre-adjust the nominal dot percentages of this new target.

**3.6.2 Print under Identical Conditions**

(1) Output this new compensated digital file to printing plates using the same screen frequencies (133 lpi and 175 lpi).

(2) Print the new validation forms on the same three types of paper (newsprint, offset, coated) using the exact same press settings (ink density, water balance, printing pressure, etc.) and environmental conditions as those used for the initial print run in Section 3.3. This controls all variables, ensuring that any change in output is solely due to the compensation algorithm.

**3.6.3 Measure the Validation Data**

After the ink is thoroughly dry, meticulously measure the printed dot area percentage on the compensated sheets. Measure each of the 25 color patches three times (as per the provided data table) for each paper type and screen frequency combination using the same spectrophotometer and procedure described in Section 3.4. This process will yield a robust validation dataset of 450 individual measurements (3 papers × 2 frequencies × 25 patches × 3 measurements).

**3.6.4 Analyze and Validate Results**

Calculate the average measured value for each of the 25 nominal dots for every paper and frequency combination, resulting in 150 validated data points (3 papers × 2 frequencies × 25 averages). Plot three curves on the same graph for comparison:

- **The Original Dot Gain Curve:** (Original nominal % vs. Original measured %) from the initial 20-point dataset (Section 3.4.1).
- **The Ideal Curve:** A straight line where y = x.
- **The Validated Compensated Output Curve:** (Compensated nominal % vs. New average measured % from the validation print).

**Key Observation:** The validated compensated output curve should lie significantly closer to the ideal curve (y=x) than the original uncompensated curve does. This visual result, derived from an independent and detailed dataset, quantitatively demonstrates the success and generalization capability of the compensation method. The improvement should be most pronounced in the mid-tone regions (40-60%).

**4. Expected Results**

(1) Successfully printed test forms with clear gradients.

(2) A dataset of 120 averaged dot gain measurements (3 papers × 2 frequencies × 20 steps).

(3) Six fitted dot gain curves (3rd-order polynomials) showing the characteristic S-shape of dot gain.

(4) Six corresponding compensation curve equations.

(5) Validation plots showing that the compensated dot output curves align significantly closer to the ideal (y=x) line than the original curves, confirming the effectiveness of the method.

**5.** **Troubleshooting**

| **Problem** | **Possible Cause** | **Solution** |
| --- | --- | --- |
| **Poor fit** (high error between curve and data points) | Data is too noisy or not S-shaped. | Ensure measurement accuracy. Check for press issues during printing. Try a different polynomial order (e.g., 2nd or 4th) and validate using goodness-of-fit metrics (e.g., R-squared). |
| **Compensation curve produces nonsensical values** (e.g., negative dots) | Overfitting or extreme values at the ends of the curve. | The model is less reliable at tone extremes (0-5%, 95-100%). Focus validation on the 10-90% range. Use the fitted curve only within the data range it was fitted to. |
| **Validation results are poor** | Press conditions not identical between initial and validation runs. | **Crucial:** Ensure all press settings (ink, water, pressure) and environmental conditions are as consistent as possible between the two print runs. |
| **MATLAB errors** | Syntax error or incorrect vector sizes. | Check that all vectors (dot_area, measured_data) are the same length (1x20). Ensure functions like polyfit are called with correct arguments. |

**6. Linkage to the Main Text**

This supporting information protocol document is directly linked to the following sections of the main paper, "*Mathematical modelling and compensation strategies for printing dot gain*":

(1) This protocol provides the detailed mathematical foundation and algorithmic implementation for the "coordinate transformation method" described in Section 2.1 and the "least squares-based curve fitting" described in Section 2.2 of the main text.

(2) This protocol offers a complete, step-by-step operational guide for the entire experimental process outlined in Section 3 of the main text, encompassing target design, printing, measurement, and data organization.

(3) The procedures described in Sections 3.2 and 3.3 of this protocol detail how the 120 averaged data points (from 360 individual measurements) mentioned in the main text were acquired.

(4) The MATLAB code and methodology provided in Sections 3.4 and 3.5 of this protocol were used to generate the fitted dot gain curves and corresponding equations presented in Fig 4, Fig 5, and Table 1 of the main text.

(5) Section 3.5 of this protocol details the generation of the compensation curves using the coordinate transformation method (resulting in the equations in Table 1 of the main text), and Section 3.6 of this protocol describes the independent validation process using a detailed 25-step target, which produced the 450 measurements used for validation and the results shown in Fig 6 and Fig 7 of the main text.

**All Numerical Findings:** All fitted equations, compensation equations, and final conclusions presented in the main text are based on the operational workflow described in this protocol.

**References**

References are identical to those cited in the main article.
